# A small dose of remifentanil pretreatment suppresses sufentanil-induced cough during general anesthesia induction: a randomized, double-blind, placebo-controlled trial

**DOI:** 10.1186/s12871-019-0836-1

**Published:** 2019-08-28

**Authors:** Wendong Lin, Jiehao Sun, Shuying Fu

**Affiliations:** 1 Department of Anesthesiology, The First Affiliated Hospital of Wenzhou Medical University, Wenzhou, 325000 China; 2 Department of Anesthesiology, The Second Affiliated Hospital and Yuying Children’s Hospital of Wenzhou Medical University, Wenzhou, 325000 China

**Keywords:** Remifentanil, Sufentanil, General anesthesia, Cough

## Abstract

**Background:**

Intravenous use of sufentanil can elicit cough. This study aimed to evaluate the inhibitory effect of pre-injection of a mall dose of remifentanil on sufentanil-induced cough during the induction of general anesthesia.

**Methods:**

This prospective, randomized, controlled trial was conducted from January 10, 2019 to March 01, 2019. A total of 100 patients undergoing elective surgery under general anesthesia were enrolled, and at last 84 patients were included and randomly allocated into two equal size groups (*n* = 42): Patients in the Remifentanil group (R group) received an intravenous infusion of remifentanil 0.3 μg/kg (diluted to 2 ml) 1 min before sufentanil injection; patients in the Control group (C group) received 2 ml of normal saline (NS) at the same time point. Injections of patients in both groups were completed within 5 s. Then, sufentanil 0.5 μg/kg was injected within 5 s and the number of coughs that occurred within 1 min after sufentanil injection were recorded. One minute after sufentanil injection, etomidate 0.3 mg/kg and cisatracurium 0.15 mg/kg were given for general anesthesia induction irrespective of the presence or absence of cough. The mean arterial pressure (MAP) and heart rate (HR) at time points just before remifentanil pretreatment administration (T0), 3 min after administration (T1), 1 min after intubation (T2), and 3 min after intubation (T3) were recorded.

**Results:**

The incidence of cough in patients in the R group and C group was 4.8 and 31%, respectively. Compared with group C, the incidence and severity of cough in group R was significantly lower (*P* < 0.01). No significant differences were observed in MAP and HR at the time of general anesthesia induction between the two groups (*P* > 0.05).

**Conclusion:**

Pretreatment with a small dose of remifentanil effectively and safely reduced the incidence and severity of cough induced by sufentanil during anesthesia induction and can be used as an alternative treatment to inhibit coughing caused by sufentanil.

**Trial registration:**

Chinese Clinical Trial Registry (ChiCTR1900020587, registered date: January 9, 2019), http://www.chictr.org.cn

**Electronic supplementary material:**

The online version of this article (10.1186/s12871-019-0836-1) contains supplementary material, which is available to authorized users.

## Background

Sufentanil has been commonly used for the induction of general anesthesia because of its strong analgesic properties [1]. However, the intravenous use of sufentanil can elicit cough [1, 2].

This side effect may increase intracranial, intraocular, and intraabdominal pressure, which may endanger patients with a cerebral aneurysm, open eye injury or abdominal aortic aneurysm.

Various agents have been used for cough suppression prior to induction of general anesthesia including: 1- intravenous opioid such as fentanyl, 2- intramuscular morphine, 3-dexmedetomidine, 4- magnesium sulfate, 5- terbutaline, 6- lidocaine, and 7- dezocine [1, 3–8].

However, because of their more or less potential additional side effects, long onset time, long duration, inconvenience to get, they were somewhat limited in clinical use.

Remifentanil is an opioid that has both short onset time and short duration and is also readily available.

The use of remifentanil for cough suppression has previously been documented [9–13]. Remifentanil has shown effective cough suppression, however, in few studies, remifentanil has been used prior to the induction of general anesthesia to prevent opioid-induced cough.

Therefore, in this study, we evaluated the effects of remifentanil on the incidence and severity of sufentanil-induced cough during the induction of general anesthesia, thereby aiming to assess whether a small dose of remifentanil could be used as an treatment to inhibit sufentanil-induced cough.

## Methods

### CONSORT guidelines

This study adheres to CONSORT guidelines.

### Participants

This study passed the ethical review by the Ethics Committee of the First Affiliated Hospital of Wenzhou Medical University (2018–150) (Wenzhou, China), and was registered at the Chinese Clinical Trial Registry (ChiCTR1900020587, January 9, 2019). Written informed consent was obtained from all patients.

**Fig. 1:**
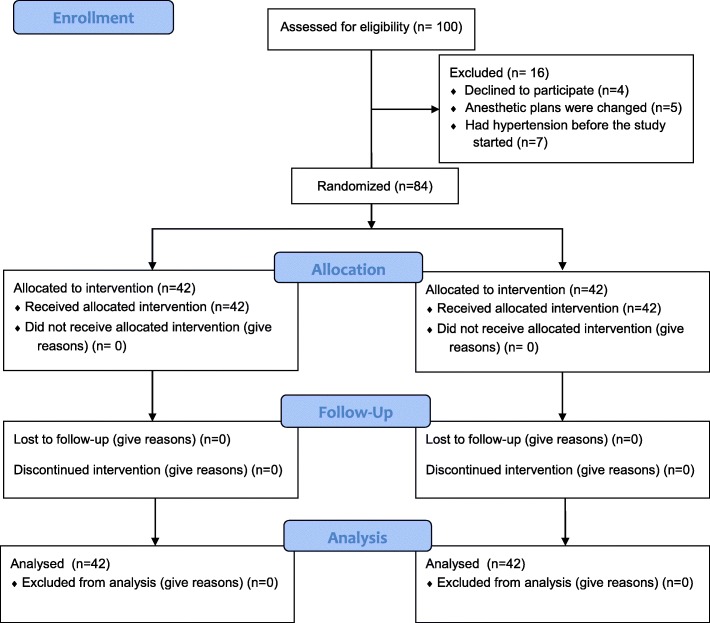
Flow of participants through the study

One hundred patients were screened, and a total of 84 patients scheduled for elective surgery under general anesthesia (aged 20–70 years), classified as ASA I or II, were selected (shown as Fig. 1).

Exclusion criteria were: smoking; Body Mass Index (BMI) over 28; increased intracranial, intraabdominal or intraocular pressure before operation; a history of respiratory diseases, such as asthma, chronic cough, and upper respiratory tract infection within two weeks; a history of other systemic diseases, including gastroesophageal reflux, hypertension, diabetes, heart disease, impaired kidney or liver function; a history of chronic administration of opioids, cough-causing medications, such as angiotensin-converting-enzyme inhibitors (ACE-inhibitors), and anti-cough medications, such as bronchodilators and steroid therapy.

### Anesthesia and research procedure

The trial was conducted from January 10, 2019 to March 01, 2019 at The First Affiliated Hospital of Wenzhou Medical University (Wenzhou, China). None of the patients received any premedication. In brief, a 20-gauge cannula was inserted into the dorsum of the hand of each patient and connected to a T-connector for drug administration. Standard ASA monitors were attached, including non-invasive arterial pressure, electrocardiography, and pulse oxygen saturation (SPO_2_). The 84 patients were randomly and equally divided into two groups: R group for remifentanil 0.3 μg/kg (diluted to 2 ml) intravenous infusion; C group for 2 ml of NS. All patients were given oxygen via a face mask (6 L/min) before the induction of anesthesia. Injections of both two groups (remifentanil or saline) were completed within 5 s. One minute after injection, each patient was given a sufentanil bolus of 0.5 μg/kg within 5 s.

The number of coughs that occurred within 1 min after sufentanil injection were recorded and the severity was graded depending on the cough times (mild, 1–2; moderate, 3–4; and severe, ≥5).

One minute after the injection of sufentanil, etomidate 0.3 mg/kg and cisatracurium 0.15 mg/kg were given for the induction of general anesthesia irrespective of the presence or absence of cough. MAP and HR immediately before remifentanil or NS administration (T0), 3 min after administration (T1), 1 min after intubation (T2), and 3 min after intubation (T3) were recorded.

Throughout the experiment, side effects of remifentanil, such as muscle rigidity or other unintended effects were also recorded.

### Statistical analysis

Sample size estimates were done using PASS 11 software (PASS, Kaysville, UT, USA). In our pilot study, the incidence of cough elicited by 0.5 μg/kg of sufentanil was 30% (6/20), which was reduced to 5% (1/20) after pretreatment with remifantanil. To achieve 80% statistical power with α = 0.05, each group would require no less than 32 patients. To account for drop-outs, we recruited 50 patients per group.

Using a computer generated random-number sequence, patients were randomly and equally divided into two groups by. Random allocation sequences were collected, participants were enrolled and assigned to interventions and outcomes were assessed. Both remifentanil (0.3 μg/kg, diluted to 2 ml) and NS were injected in a volume of 2 ml. After assignment, both participants and investigators were blinded to interventions.

Data were analyzed by SPSS software 22.0 (IBM Corp, Armonk, NY, USA). The normality of measurement data distribution was tested by the Shapiro-Wilk test. Measurement data of normal distribution (age, BMI, etc.) were presented as the mean ± standard deviation and were analyzed by Student’s t test. Measurement data of non-normal distribution were presented as the median (Q1, Q3) and analyzed by the Mann–Whitney U test. Categorical data were presented as absolute and relative effect sizes, and assessed by the Chi-square test or Fisher’s exact test as appropriate. Grade data (the severity of coughs) were analyzed by Mann–Whitney U test. Continuous variables at different time points were compared by using repeated measures design analysis of variance. *P* < 0.05 was considered statistically significant.

## Results

### Demographic characteristics

This trial was conducted from January 10, 2019 to March 01, 2019. A total of 100 eligible patients were screened among which 84 patients participated in our study and were included in the final analyses (four patients declined participation; seven patients had unacceptable hypertension before the start of the study; the anesthetic plans of five patients changed before the study started) (Fig. 1).

ASA physical status and demographic characteristics, including gender, age, and the BMI of patients in the two groups were similar (*P* > 0.05, Table 1).

**Table 1:** Demographic data

Group	Gender (M/F)	Age (yr)	ASA class (I/II)	BMI (kg/m^2^)
Group C(n = 42)	16/26	43.6 ± 11.4	16/26	22.5 ± 1.9
Group R(n = 42)	18/24	45.6 ± 11.0	11/31	22.9 ± 2.9
P value	0.657	0.414	0.243	0.449

Values are expressed as the mean ± standard deviation or as number of cases.

No statistical differences were observed between groups in gender, age, ASA class or BMI.

R = remifentanil, C = control; M = male, F = female; ASA = American Society of Anesthesiologists physical status; BMI = body mass index.

### Incidence and severity of cough

The incidence in group C was 31% and in group R was 4.8%. Both the incidence and severity of cough in Group R were significantly lower than those in Group C (p < 0.01, Table 2).

**Table 2:** Incidence and severity of sufentanil induced cough

Groups	Incidence of cough (n(%))	Severity of cough (n(%))			
		None	Mild	Moderate	Severe
Group C(n = 42)	31	69	7.1	4.8	19
Group R(n = 42)	4.8	95.2	2.4	0	2.4
P value	0.002^*^	0.002^*^			

Values are expressed as frequency.

Both the incidence and severity of cough in patients in Group R were significantly lower compared to those in patients in Group C (*P* < 0.01).

R = remifentanil; C = control. The severity of cough was graded as mild (1, 2), moderate (2, 3), or severe (> 5) based on the number of cough observed in 60s after sufentanil injection. *Indicates *P* < 0.01 compared with patients in Group C.

### Hemodynamic data changes

There was no statistical difference in the data of HR and MAP between two groups at four corresponding time points (p > 0.05, Table 3 and Table 4).

**Table 3:** Changes of MAP

Groups	MAP (mm Hg)			
	T0	T1	T2	T3
Group C	94.9 ± 13.8	76.2 ± 12.9	93.9 ± 20.4	82.0 ± 17.0
Group R	99.0 ± 12.7	78.7 ± 13.3	95.0 ± 16.5	84.7 ± 15.5
P value	*P* = 0.322

**Table 4:** Changes of HR

Groups	HR (bpm)			
	T0	T1	T2	T3
Group C	80.0 ± 18.2	68.6 ± 16.9	77.0 ± 19.0	67.7 ± 15.2
Group R	78.9 ± 11.3	67.9 ± 11.0	77.8 ± 13.1	70.5 ± 10.9
*P* value	*P* = 0.886			

Values are expressed as mean ± standard deviation.

There were no significant differences in ether MAP or HR between the two groups.

R = remifentanil, C = control; T0, time before administration of remifentanil or normal saline; T1: 3 min after administration; T2: 1 min after intubation; T3: 3 min after intubation; MAP: mean arterial pressure; HR: heart rate.

### Side effects or unintended effects

In both groups, none of the patients showed signs of apnea, muscle rigidity or other unintended effects.

## Discussion

In this study, we demonstrated that pretreatment with remifentanil at a small dose of 0.3 μg/kg reduced the incidence and severity of sufentanil-induced cough without influencing the hemodynamics during anesthetic induction. The incidence of cough was 31% in patients in group C and decreased to 4.8% in patients in Group R.

Coughing following the administration of opioid drugs during general anesthesia induction is often reported. In this study, the incidence of cough induced by sufentanil was 31%. In an study by Agarwal et al. [2] sufentanil 0.3 μg/kg injected over 5 s elicited cough in 15.8% of patients, while in another study by Li et al. [14] the incidence of cough was 37% after the injection of sufentanil 0.5 μg/kg within 3 s. With a high dose of sufentanil (1 μg/kg), the incidence of sufentanil-induced cough could be up to 45.8% [1]. The various incidence among different studies might be due to the different doses of sufentanil used and the differences in concentrations, administration rate, race and age [1, 15].

Various mechanisms have been proposed to explain opioid-induced cough. A plumonary chemoreflex mediated by either vagal C-fiber receptors close to pulmonary vessels or irritant receptors may play a role in opioid-induced cough [16]. Opioid-induced histamine release [17] and muscle rigidity leading to sudden closure of the vocal cords or supraglottic obstruction by soft tissue [18] may be another possible casual factor for cough. In addition, in several studies, it was demonstrated that opioid receptor dualism might be an important mechanism of opioid-induced cough [3, 8, 19].

Various pretreatments with drugs, such as lidocaine, terbutaline, dezocine, dexmedetomidine, and magnesium sulfate have been reported to reduce the incidence of opioid-induced cough [1, 5, 6, 8, 20], however these drugs might add side effects and potential risks. In a study by He et al. [5], an injection with dexmedetomidine 0.5 μg/kg or 1.0 μg/kg over 10 min inhibited cough induced by the subsequent fentanyl (4 μg/kg) intravenous injection. However, this dose of dexmedetomidine has the potential to cause bradycardia and hypotension. Furthermore, an injection that is too slow may cause an issue. In another study [1], 30 or 50 mg/kg of MgSO_4_ inhibited cough induced by a subsequent injection with 1.0 μg/kg sufentanil, however several patients dropped out of the study due to an obvious burning sensation during the injection of MgSO_4_. In addition, the injection of MgSO_4_ could increase plasma levels of magnesium. Furthermore, inhalation of terbutaline (5 mg in 2 ml NS, via a jet nebulizer) fifteen minutes before bolus fentanyl (5 μg/kg, iv) has been reported to inhibit cough [6], however the inaccessibility of terbutaline, long operating time, and complicated operations are some limitations of this approach. The injection of lidocaine 0.5 mg/kg has been proven effective to suppress fentanyl-induced cough [20], however the inhibition of cardiac function may be a potential risk. Dezocine 0.1 mg/kg 2 min prior to the administration of intravenous sufentanil (0.5 μg/kg) can also effectively suppress fentanyl-induced cough [8], however in short surgeries, the excessive sedation time of this dose of dezocine may be a problem.

In several other studies, a pre-emptive small dose of the opioids was used to inhibit opioid-induced cough, which could avoid the additional side effects or potential risks of other types of drugs. Hung et al. [4] reported that a pre-emptive small dose of fentanyl (25 μg) significantly reduced cough induced by a subsequent injection of 125 μg fentanyl, and Phua et al. [3] showed that intramuscular morphine pretreatment 1 h before general anesthesia induction could also decrease the incidence of fentanyl-induced cough. However, either fentanyl or morphine has limitations because of long onset or long duration time.

In the current study, we found that remifentanil, an opioid that is readily available, with both short onset and duration time, could decrease the incidence and severity of sufentanil-induced cough during anesthetic induction.

It is not yet clear how small doses of opioids inhibit the cough caused by opioids themselves. In a study by Hung et al. [4], a pre-emptive small dose of fentanyl (25 μg) significantly reduced cough induced by a subsequent injection with 125 μg fentanyl, and this phenomenon was thought to be associated with the lower plasma concentration fluctuation of fentanyl.

However, in the current study, we found that a pre-emptive small dose of 0.3 μg/kg remifentanil reduced cough induced by a subsequent injection with 0.5 μg/kg sufentanil. However, what was used as a pretreatment was another opioid, remifentanil, not sufentanil itself. Hence, the theory of fluctuations in the sufentanil plasma concentration mentioned above seems unlikely.

We hypothesized that opioid receptor dualism may be a possible mechanism to partly explain this phenomenon. One of the common and useful side-effects of opioid analgesics is suppression of the cough reflex, which is the basis of their use in oral cough suppressants [3]. Sufentanil, fentanyl, and remifentanil infusion before recovery from general anesthesia have previously been reported to suppress coughing during extubation [9, 21, 22]. Opioids may inhibit the cough reflex by a direct effect on the cough center in the medulla, at doses lower than those required for analgesia [3]. Therefore, we suggested that the anti-tussive effect of pre-emptive remifentanil in the present study might be related to a centrally-acting effect. The low priming dose of remifentanil might initially exert a central anti-tussive effect and then inhibit the cough-inducing effect of a subsequent large dose of sufentanil. This hypothesis was partly supported by the study of Phua et al. [3] in which the incidence of fentanyl-induced cough was reduced by intramuscular morphine pretreatment 1 h before general anesthesia induction. However, this speculation lacks definitive evidence, and therefore additional studies are warranted to verify this hypothesis, and to reveal the underlying mechanism of action.

Given that remifentanil has a respiratory depressive effect, possible apnea-related absence of cough after remifentanil administration must be considered. It is also possible that the dose of remifentanil caused a transient apnea that resulted in an absence of coughing. For example, Remifentanil took 50–60 s to reach the effect point, and the apnea took 40–50 s, which partially or even completely covered the observation time after sufentanil administration.

Remifentanil may induce muscle rigidity and blood pressure and HR decline [15]. In a study performed by Shen et al. [15], three patients in the rernifenranil group showed muscle rigidity. and one patient had significant bradycardia that required treatment with atropine. However, in our study, none of the patients showed such adverse reactions, which might be due to the very low dose of remifentanil that was used.

Our study has several limitations. Few studies have shown the number doses of remifentanil that are used to suppress cough caused by opioids, therefore, we used a dose of remifentanil that was close to the dose of fentanyl used in the study of Hung et al. [4] in which a pre-emptive small dose of fentanyl (25 μg) was used to reduce cough induced by a subsequent injection with125 μg fentanyl. Thus, we used a single dose of remifentanil, and therefore do not know whether different doses, such as 0.1 μg/kg, 0.2 μg/kg, 0.4 μg/kg would be effective in suppressing the sufentanil-induced cough reflex. Additional studies will need to be performed to explore the relationship between dose and the cough suppressing effect of remifentanil.

## Conclusion

In conclusion, pretreatment with a small dose of remifentanil effectively and safely suppresses the incidence and severity of sufentanil-induced cough during the induction of general anesthesia, and may be used as an alternative treatment to inhibit sufentanil-induced cough.

## Additional file

Additional file 1: Original data. This is the dataset supporting the conclusions of this article, including demographic characteristics, severity of cough, hemodynamic changes and side effects or unintended effects. (XLSX 16 kb)

## Data Availability

The main data have been presented in the article and its additional file (Additional file 1). All the other data and materials supporting the conclusions of this article are available from the corresponding author on reasonable request.

## Abbreviations

ACE: Angiotensin-converting-enzyme
ASA: American Society of Anesthesiologists
BMI: Body mass index
HR: Heart rate
IV: Intravenous
MAP: Mean arterial pressure
NS: Normal saline
SPO_2_: Pulse oxygen saturation
SPSS: Statistical Product for Social Sciences

## References

[1] An L, Gui B, Su Z, Zhang Y, Liu H (2015). Magnesium sulfate inhibits sufentanil-induced cough during anesthetic induction. Int J Clin Exp Med.

[2] Agarwal A, Gautam S, Nath SS, Gupta D, Singh U (2007). Comparison of the incidence and severity of cough induced by sufentanil and fentanyl: a prospective, randomised, double-blind study. ANAESTHESIA..

[3] Phua Wee Thuan, Teh Boon Teck, Jong Winston, Lee Tat Leang, Tweed William A. (1991). Tussive effect of a fentanyl bolus. Canadian Journal of Anaesthesia.

[4] Hung KC, Chen CW, Lin VCH, Weng HC, Hsieh SW (2010). The effect of pre-emptive use of minimal dose fentanyl on fentanyl-induced coughing. ANAESTHESIA [Article].

[5] He L, Xu J, Dai R (2012). Dexmedetomidine reduces the incidence of fentanyl-induced cough: a double-blind, randomized, and placebo-controlled study. UPSALA J MED SCI.

[6] Lui PW, Hsing CH, Chu YC (1996). Terbutaline inhalation suppresses fentanyl-induced coughing. CANADIAN JOURNAL OF ANAESTHESIA-JOURNAL CANADIEN D ANESTHESIE. [Article]..

[7] Guler G, Aksu R, Bicer C, Tosun Z, Boyaci A (2010). Comparison of the effects of ketamine or lidocaine on fentanyl-induced cough in patients undergoing surgery: a prospective, double-blind, randomized, placebo-controlled study. CURRENT THERAPEUTIC RESEARCH-CLINICAL AND EXPERIMENTAL. [Article]..

[8] Liu XS, Xu GH, Shen QY, Zhao Q, Cheng XQ, Zhang J, et al. Dezocine prevents sufentanil-induced cough during general anesthesia induction: a randomized controlled trial. PHARMACOL REP [Journal Article; Randomized Controlled Trial; Research Support, Non-US Gov't] 2015 2015-02-01;67(1):52–55. 10.1016/j.pharep.2014.08.004 25560575

[9] Cho HB, Kim JY, Kim DH, Kim DW, Chae YJ. Comparison of the optimal effect-site concentrations of remifentanil for preventing cough during emergence from desflurane or sevoflurane anaesthesia. J INT MED RES. [Comparative Study; Journal Article; Randomized Controlled Trial]. 2012 2012-01-20;40(1):174–183. 10.1177/147323001204000118 22429357

[10] Jun NH, Lee JW, Song JW, Koh JC, Park WS, Shim YH. Optimal effect-site concentration of remifentanil for preventing cough during emergence from sevoflurane-remifentanil anaesthesia. ANAESTHESIA. [Journal Article; Randomized Controlled Trial]. 2010 2010-09-01;65(9):930–935. 10.1111/j.1365-2044.2010.06450.x 20645945

[11] Kim H, Choi SH, Choi YS, Lee JH, Kim NO, Lee JR. Comparison of the antitussive effect of remifentanil during recovery from propofol and sevoflurane anaesthesia. ANAESTHESIA. [Comparative Study; Journal Article; Randomized Controlled Trial]. 2012 2012-07-01;67(7):765–770. 10.1111/j.1365-2044.2012.07136.x 22519849

[12] Lee B., Lee J.-R., Na S. (2009). Targeting smooth emergence: the effect site concentration of remifentanil for preventing cough during emergence during propofol–remifentanil anaesthesia for thyroid surgery. British Journal of Anaesthesia.

[13] Yoo JY, Kim JY, Kwak HJ, Lee DC, Kim GW, Lee SY, et al. - Effect-site concentration of remifentanil for preventing cough during emergence in elderly patients undergoing nasal surgery: a comparison with adult patients. - Clin Interv Aging 2016 Sep 12;11:1247–1252. doi: 10.2147/CIA.S108705 . eCollection 2016.(- 1178-1998 (Electronic); - 1176-9092 (Linking)). 10.2147/CIA.S108705 PMC5026220 27672319

[14] Li J, Li K. Effects of pre-inhalation of salbutamol on cough reflex induced by Sufentanil. In: Sadeghian SH, Mehta U, Rajbongshi P, ^editors. AER-Advances in Engineering Research; 2016. p. 237–9.

[15] Shen J, Xu J, Zhou Z, Liu H, Yang J (2008). Effect of equivalent doses of fentanyl, Sufentanil, and remifentanil on the incidence and severity of cough in patients undergoing abdominal surgery: a prospective, randomized, double-blind study. Current therapeutic research-clinical and experimental [article].

[16] Böhrer H, Fleischer F, Werning P. Tussive effect of a fentanyl bolus administered through a central venous catheter. ANAESTHESIA. 1990 1990-01-01;45(1):18–21. 10.1111/j.1365-2044.1990.tb14496.x 2316832

[17] Karlsson J. A., Sant'Ambrogio G., Widdicombe J. (1988). Afferent neural pathways in cough and reflex bronchoconstriction. Journal of Applied Physiology.

[18] Benthuysen JL, Smith NT, Sanford TJ, Head N, Dec-Silver H. Physiology of alfentanil-induced rigidity. ANESTHESIOLOGY. 1986 1986-04-01;64(4):440–446. 10.1097/00000542-198604000-00005 3008595

[19] Ai Q, Hu Y, Wang Y, Wu S, Qin Z, Wang J, et al. Pentazocine pretreatment suppresses fentanyl-induced cough. PHARMACOL REP. [Journal Article; Randomized Controlled Trial]. 2010 2010-07-01;62(4):747–750. 10.1016/s1734-1140(10)70333-9 20885016

[20] Pandey CK, Raza M, Ranjan R, Singhal V, Kumar M, Lakra A (2005). Intravenous lidocaine 0.5 mg.Kg(−1) effectively suppresses fentanyl-induced cough. CANADIAN JOURNAL OF ANAESTHESIA-JOURNAL CANADIEN D ANESTHESIE [Article].

[21] Yoo YC, Na S, Jeong JJ, Choi EM, Moon BE, Lee JR (2011). Dose-dependent attenuation by fentanyl on cough during emergence from general anesthesia. ACTA ANAESTH SCAND [Article].

[22] Lee JY, Lim BG, Park HY, Kim NS. Sufentanil infusion before extubation suppresses coughing on emergence without delaying extubation time and reduces postoperative analgesic requirement without increasing nausea and vomiting after desflurane anesthesia. Korean J Anesthesiol [Journal Article] 2012 2012-06-01;62(6):512–517. 10.4097/kjae.2012.62.6.512 PMC3384787 22778885

